# Cardiac “potential energy” estimation: ambiguous and subjective

**DOI:** 10.1152/japplphysiol.00761.2023

**Published:** 2024-03-07

**Authors:** June-Chiew Han, Toan Pham, Andrew J. Taberner, Kenneth Tran

**Affiliations:** ^1^Auckland Bioengineering Institute, The University of Auckland, Auckland, New Zealand; ^2^Department of Engineering Science and Biomedical Engineering, The University of Auckland, Auckland, New Zealand

With each ejecting beat, the heart converts biochemical energy into external mechanical work (*W*). It would seem obvious, then, that its metabolic energy expenditure should be correlated with the external work (*W*) it performs, i.e., *Energy* ∝ *W* (1–4). However, such has not been commonly regarded to be the case. Rather, cardiac energy expenditure is widely accepted to be correlated with the sum of *W* and an additional energy term that is referred to as “potential energy” (*PE*), i.e., *Energy* ∝ *W* + *PE*. The term *PE* is sizable: its magnitude is as large as *W* under ejecting/shortening (or work-loop) contractions, and it constitutes entirely the energy term under isovolumic/isometric contractions.

This concept was formulated in the 1980s by Suga and colleagues (5–7) using data from the isolated canine heart and has since been used to interpret data from a variety of species including rodent and human, and from intact hearts and isolated muscle preparations. *PE* is estimated from the ventricular pressure-volume (*P*-*V*) or muscle force-length (*F*-*L*) plane, defined by a rather specific “triangle” area. Suga asserted that *PE* is potential energy that could be converted into *W* or, else, liberated as heat. We have thus directly measured both heat and *W* in our experiments under both isometric and work-loop contractions (8–10).

In this Viewpoint, we reflect on our collective studies and our understanding of Suga’s pressure-volume area (PVA) concept (8, 11, 12) to illustrate both the conceptual vagueness and the methodological uncertainty in the estimation of *PE*. This Viewpoint is an appeal to researchers in this field to discontinue using the potential energy concept to infer cardiac efficiency. Hereinafter, we label *PE* as *U* to signify that its estimation is often uncertain.

## ESTIMATION OF *U* UNDER ISOMETRIC CONTRACTIONS

**Figure 1.**
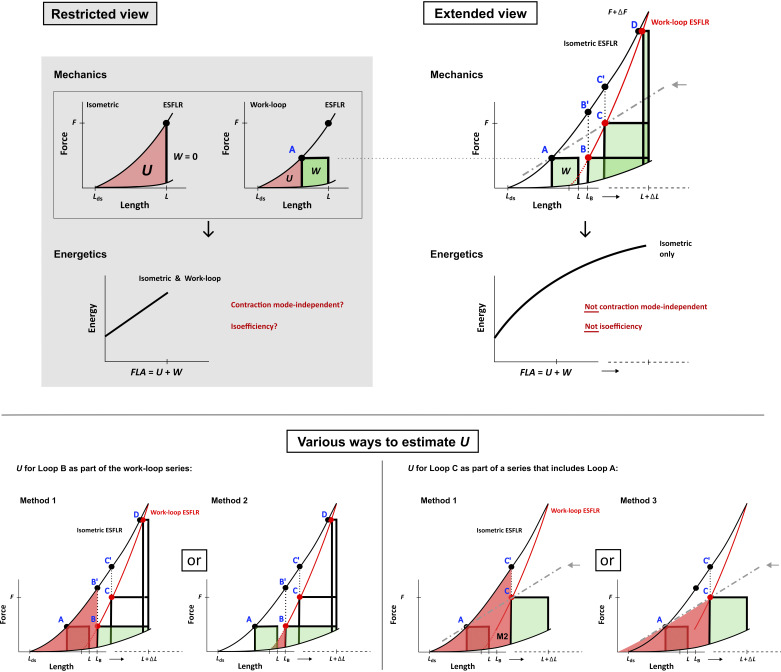
Cardiac end-systolic force-length relation (ESFLR) is contraction mode-dependent: this is the basis for the ambiguity in the estimation of *U* under work-loop contractions, which has consequences on understanding cardiac energetics. Cardiac force-length relation is the 1-dimensional equivalent of cardiac pressure-volume relation. This figure is partitioned into 3 panels: *top left*: restricted view of the force-length (*F*-*L*) axis and energy-force-length area (*FLA*) axis; *top right*: extended view of the force-length (*F* + ΔF and *L* + Δ*L*) and energy-*FLA* axis (as indicated by the arrow pointing to the positive *x*-axis); and *bottom*: numerous estimates of *U* for a given work-loop. *Top left:* cardiac muscle can contract in an isometric manner (where external work, *W*, is zero) or with shortening, where muscles perform force-length work-loop and produce external work (*W*; colored in green). *U* is colored in magenta for both contraction modes and occupies the area below the isometric end-systolic force-length relation (ESFLR). The ESFLR is taken to be contraction mode-independent, i.e., that defined from isometric contractions is equal to that defined from work-loops. This assumption holds only under limited conditions of low preload (or muscle length), as plotted in the “Restricted View.” *Top right*: when the length axis is expanded, i.e., by stretching the muscle, as plotted in the “Extended View,” then the isometric ESFLR (black curve) diverges from the work-loop ESFLR (red curve), i.e., this difference renders the calculation of *U* uncertain, as the end-systolic points of the work-loops do not overlay and align with the isometric ESFLR (i.e., the red circle is not the same as the black circle). Cardiac ESFLR is contraction mode-dependent. Under physiological conditions where a series of work-loops arises in response to simultaneously increasing preload and afterload, experimentalists typically fit a polynomial to the end-systolic points to form a ESFLR. If *loops A* and *C* are 2 of a series of those work-loops, the resulting ESFLR (broken gray line; indicated by the gray arrow) clearly resembles neither the isometric ESFLR nor the work-loop ESFLR. B’ and C’ are the corresponding isometric end-systolic points obtained at the end-systolic length of *loops B* and *C*, respectively. On the Energetics space, when *U* can be calculated (under limited conditions, i.e., at low preloads/lengths), the energy consumed by muscle is taken to be proportional to pressure-volume area (*PVA*; or *FLA*), which sums *U* and *W*, and has been widely assumed to be independent of the mode of contraction (shown in the Restricted View). The inverse slope of this linear relation is taken as a measure of efficiency, i.e., Suga’s isoefficiency, which is contraction mode-independent and load-independent. At a larger view (*right*; shown in the Extended View), where muscle length is increased, the linear relation between energy and FLA is, in fact, not linear and can only be obtained under isometric contractions (where *U* can be calculated over the entire extent of the ESFLR). *Bottom*: the Extended View of the *F*-*L* axis is transcribed from the *top right* and simplified to illustrate numerous estimations of *U* (colored in magenta) for *loop B* (*left*) and, likewise, numerous estimations of *U* for *loop C* connected with *loop A* (*right*). *Method 1* requires the isometric ESFLR (black line) as the upper boundary of the area for *U*; *Methods 2* and *3* require the work-loop ESFLR (red lines), the relation of which depends on the fitting to collected end-systolic points. *Method 2* for *loop C* is labeled “M2” under the area for *U*, as consistent with that of *Method 2* for *loop B*.

*U* is estimated as the triangle area at the bottom left corner of the *P*-*V* or *F*-*L* plane (the area colored magenta in Fig. 1). Its upper boundary is the isometric end-systolic *F*-*L* relation (ESFLR) obtained by fitting to a series of isometric contractions at different muscle lengths. The fitted isometric ESFLR intersects the dead-space length (*L*_ds_) on the *x*-axis: a value that is often challenging to determine experimentally and, hence, is often estimated by extrapolation of the ESFLR to the *x*-axis. Isolated muscle preparations with their length reduced to the point where negligible macroscopic force is observed can satisfactorily allow measurement of *L*_ds_. Isolated preparations, both whole-heart and muscle, can be stretched to the optimal volume or length where maximum pressure or force is attained to estimate the maximum *U*. A complete range of *U*, at any given muscle length, from the minimal to the maximal extent of the isometric ESFLR can thus be estimated.

## AMBIGUOUS ESTIMATION OF *U* UNDER WORK-LOOP CONTRACTIONS

The estimation of *U* described above for isometric contractions cannot be readily applied to work-loop contractions where muscle shortens and produces external *F*-*L* work (*W*). We illustrate this in Fig. 1, *top right*, comparing *loop* A to *loop B*, where the afterload is held constant while initial volume or muscle length is increased as achieved experimentally (8, 13). The end-systolic point of *loop B* deviates from the isometric ESFLR, making the estimation of *U* for *loop B* ambiguous. Thus *U* could be estimated either (*method 1*) by the triangle area *L*_ds_-B’-*L*_B_, where B’ is the isometric contraction that matches the end-systolic length (*L*_B_) of *loop B*, thereby meeting the requirement that the upper boundary of *U* is the isometric ESFLR and intersect *L*_ds_ (as illustrated in Fig. 1, *bottom*), or (*method 2*) by violating this requirement, where the upper boundary of *U* is not the isometric ESFLR but is now the fitted work-loop ESFLR (red line) and does not intersect *L*_ds_. The former (14) and the latter (15) methods of estimation have both been adopted in the literature. Clearly, these methods cannot both provide the same estimate of *U*: the latter yields a *U* that is only a tiny fraction of that estimated using the former (Fig. 1, *bottom right*).

The ambiguity compounds when we consider the case where preload and afterload simultaneously increase to produce a series of loops, i.e., *loop A* and *loop C* are part of the series, as obtained experimentally (16–18) and clinically (19). One would have to choose not just between one of the two methods described for *loop B* to estimate the *U* for *loop C* but with now an additional method (*method 3*; Fig. 1, *bottom left*) that fits an ESFLR to the end-systolic points of *loop A* and *loop C* (broken gray line). Additional estimates of *U* for *loop C* can arise from various regression models that can be used to describe the broken gray line. In the literature, nonlinear fitting models may need to be chosen to obtain positive values of *L*_ds_ or dead-space volume (*V*_0_) particularly when a linear regression would otherwise result in an extrapolation to the negative side of the volume axis yielding physiologically nonsensical estimates of *V*_0_ (16–18). Given that *L*_ds_ (or *V*_0_) and *U* are both susceptible to the fitting regression models, that can lead to numerous ambiguous estimates of *U*.

## WHAT REMAINS?

It has become clearer that Suga based his concept of estimating *U* from experiments conducted over low initial ventricular volumes (or initial muscle lengths; i.e., *loop A*), a limitation that he recognized when he stated that his preparations could not meet the challenge at high preloads and suffered from frequent arrhythmias (20). His concept is limited to low preloads because when muscle length is increased, work-loop end-systolic points diverge from the isometric ESFLR due to contraction mode-dependency and, hence, produce an inconsistency in the estimation of *U*, which assumes a single, contraction mode-independent, ESFLR. We assume that he was aware of the divergence of isovolumic and work-loop ESFLRs when he redrew the *P*-*V* diagram of Otto Frank to illustrate contraction mode-dependency (21).

We do not intend to diminish the value of the academic accomplishments of Suga over the 1980s to 2000s. The field at that time was in an indecisive state of affairs, loaded with conflicting views on contraction mode-dependency of cardiac ESFLR (8, 12, 22–24), and on the load-dependency of cardiac efficiency. The latter birthed the concept of “isoefficiency,” where Suga used the ratio of the sum of *U* and *W* [i.e., force-length area (FLA)] to oxygen consumption (i.e., the inverse slope of the linear energy-FLA relation; Fig. 1) to quantify a constant value for “cardiac efficiency,” which is inconsistent with the conventional definition of cardiac mechanical efficiency where the numerator consists solely of *W* (11, 25, 26).

We have recently resolved the contradiction by showing and re-confirming that the cardiac ESFLR is dependent on the mode of contraction (8). We revealed that cardiac ESFLR appears to be independent only under certain, limited, conditions where either the preload is low (or low muscle length; *loop A*) or the afterload is high (approaching isometric force at a given preload; *loop D* in Fig. 1, *top right*). We further demonstrated that contraction mode-dependency also holds on the energy-force plane (27), reinforcing that the energy-FLA (or PVA) relation, which appears to be load independent is, in fact, load dependent. At long muscle lengths, the linear energy-FLA relation obtained under isometric contractions, where *U* can be fully estimated, has been shown to be nonlinear (28) as drawn in Fig. 1, *top right*, under “Energetics,” and such nonlinearity is supported by published experimental data (29–31). Thus Suga’s isoefficiency is drawn from a quite limited range of loading conditions and does not hold when the mode dependency of cardiac ESFLR becomes apparent. Cardiac mechanical efficiency is both afterload and preload dependent (2). This evidence collectively annuls the supposed estimate of *U* and, consequently, the physiological basis of Suga’s isoefficiency.

What remains of Suga’s framework is the calculation of *U* under only isometric contractions: it is not useful during work-loop contractions where the estimation of *U* is ambiguous and subjective. If the field is to continue estimating *U*, this will sustain an ambiguous assessment and understanding of cardiac energetics, in particular cardiac activation energy (32) and cardiac efficiency.

## GRANTS

This study was supported by the Health Research Council of New Zealand through Sir Charles Hercus Health Research Fellowship Grants (20/011 and 21/116; awarded to J.-C.H. and K.T., respectively), Explorer Grant (21/758, awarded to J.-C.H.) and Emerging Researcher First Grant (21/653, awarded to T.P.), the Royal Society of New Zealand through Marsden Project Grant (MFP-UOA2206, awarded to J.-C.H.) and James Cook Research Fellowship (awarded to A.J.T.), and the Heart Foundation of New Zealand through Project Grant (1929, awarded to J.-C.H.) and Research Fellowship Grant (1896, awarded to T.P.).

## DISCLOSURES

No conflicts of interest, financial or otherwise, are declared by the authors.

## AUTHOR CONTRIBUTIONS

J.-C.H. conceived and designed research; J.-C.H. performed experiments; J.-C.H. analyzed data; J.-C.H., T.P., A.J.T. and K.T. interpreted results of experiments; J.-C.H. prepared figures; J.-C.H. drafted manuscript; J.-C.H., T.P., A.J.T., and K.T. edited and revised manuscript; J.-C.H, T.P., A.J.T., and K.T. approved final version of manuscript.
